# Changes in the chemical compositions and biological properties of kombucha beverages made from black teas and pineapple peels and cores

**DOI:** 10.1038/s41598-023-34954-7

**Published:** 2023-05-15

**Authors:** Ly Tu Phung, Haruthairat Kitwetcharoen, Nuttaporn Chamnipa, Nongluck Boonchot, Sudarat Thanonkeo, Patcharaporn Tippayawat, Preekamol Klanrit, Mamoru Yamada, Pornthap Thanonkeo

**Affiliations:** 1 Department of Biotechnology, Faculty of Technology, Khon Kaen University, Khon Kaen, 40002 Thailand; 2 Walai Rukhavej Botanical Research Institute, Mahasarakham University, Maha Sarakham, 44150 Thailand; 3 Faculty of Associated Medical Sciences, Khon Kaen University, Khon Kaen, 40002 Thailand; 4 Fermentation Research Center for Value Added Agricultural Products (FerVAAPs), Khon Kaen University, Khon Kaen, 40002 Thailand; 5 Department of Biological Chemistry, Faculty of Agriculture, Yamaguchi University, Yamaguchi, 753-8315 Japan; 6 Research Center for Thermotolerant Microbial Resources, Yamaguchi University, Yamaguchi, 753-8315 Japan

**Keywords:** Biotechnology, Microbiology

## Abstract

Several raw materials have been used as partial supplements or entire replacements for the main ingredients of kombucha to improve the biological properties of the resulting kombucha beverage. This study used pineapple peels and cores (PPC), byproducts of pineapple processing, as alternative raw materials instead of sugar for kombucha production. Kombuchas were produced from fusions of black tea and PPC at different ratios, and their chemical profiles and biological properties, including antioxidant and antimicrobial activities, were determined and compared with the control kombucha without PPC supplementation. The results showed that PPC contained high amounts of beneficial substances, including sugars, polyphenols, organic acids, vitamins, and minerals. An analysis of the microbial community in a kombucha SCOBY (Symbiotic Cultures of Bacteria and Yeasts) using next-generation sequencing revealed that *Acetobacter* and *Komagataeibacter* were the most predominant acetic acid bacteria. Furthermore, *Dekkera* and *Bacillus* were also the prominent yeast and bacteria in the kombucha SCOBY. A comparative analysis was performed for kombucha products fermented using black tea and a fusion of black tea and PPC, and the results revealed that the kombucha made from the black tea and PPC infusion exhibited a higher total phenolic content and antioxidant activity than the control kombucha. The antimicrobial properties of the kombucha products made from black tea and the PPC infusion were also greater than those of the control. Several volatile compounds that contributed to the flavor, aroma, and beneficial health properties, such as esters, carboxylic acids, phenols, alcohols, aldehydes, and ketones, were detected in kombucha products made from a fusion of black tea and PPC. This study shows that PPC exhibits high potential as a supplement to the raw material infusion used with black tea for functional kombucha production.

## Introduction

Kombucha is one of the most popular functional beverages consumed worldwide. It is a fermented beverage made by microbial fermentation of sweetened tea using mixed consortia of bacteria and yeasts, known as a symbiotic culture of bacteria and yeasts (SCOBY)^1^. Kombucha has been renowned for its health benefits due to its rich contents of various beneficial metabolites, which are not only liberated from raw material but also generated during fermentation, such as polyphenols, organic acids, vitamins, amino acids, minerals, proteins, and hydrolytic enzymes^1–5^. The content of the main active ingredients in kombucha products varied depending on the type of tea, fermentation conditions, and microbial community in kombucha SCOBY. For instance, the total phenolic content (TPC) of kombucha prepared from green, oolong, and black teas were 1.248, 1.011, and 0.455 mg gallic acid/mL kombucha, respectively. On the other hand, black tea kombucha exhibits higher organic acids content, such as gluconic acid (70.11 g/L), acetic acid (11.15 g/L), D-saccharic acid 1,4-lactone (DSL) (5.23 g/L), succinic acid (3.05 g/L), glucuronic acid (1.58 g/L), and ascorbic acid (0.70 g/L) than green and oolong teas^6^. A study by Jakubczyk et al.^7^ demonstrated that red tea kombucha contained 242.50 mg/L of total flavonoid content (TFC), which was higher than green tea (181.30 mg/L), black tea (126.70 mg/L), and white tea (111.60 mg/L) kombuchas. As shown in literature reviews, kombucha possesses significant health benefits, including antioxidant, antimicrobial, anti-inflammatory, and anticancer effects, as well as other capacities to support the immune system and reduce health problems related to diabetes, digestion, and cardiovascular diseases^5,8–13^. However, it should also be concerned that excessive consumption of kombucha products can cause lactic acidosis, specifically in immunocompromised people, heavy alcohol drinkers, and pregnant women^14,15^. Kombucha has also been implicated in hyponatremia, toxic and cholestatic hepatitis, and anti-Jo1 myositis^16–19^. Due to the increasing consumer demand for functional drinks, kombucha is believed to be one of the most promising options, and sales are projected to reach 10.45 billion dollars in 2027^14,20^.

Kombucha is typically produced from black tea (*Camellia sinensis*) and sugar, mainly sucrose. However, different types of tea, such as green, oolong, white, rooibos, and Pu’er teas^6,7,12,21,22^, and different sugars and sweetening agents, such as lactose, glucose, fructose, coconut sugar, molasses, and aspartame^23–25^, have also been evaluated as the main ingredients for kombucha production, which provides different chemical compositions, organoleptic characteristics, biological properties, and health effects from the kombucha products. Apart from the main ingredients, alternative raw materials have been reported to replace the traditional components partially or completely and enhance the biological properties and beneficial health effects, including wheatgrass^26^, coconut water^27^, banana peel and nettles^28^, oak leaf^29^, guava leaf^30^, snake or salak fruit^31^, olive leaf and honey^32^, pineapple, apple, and pomegranate juices^33^, papaya^34^, malvavisco or wax mallow flowers^35^, and mint^36^. Furthermore, the use of other microorganisms to replace the kombucha SCOBY, such as *Trametes versicolor* and *Lentinula edodes*, has also been investigated^37^.

Over the years, the global consumption of fresh and processed pineapple has increased because it has various health benefits. Industrial pineapple production generates many pineapple byproducts, such as the peels, cores, leaves, and stems, which account for approximately 50% (w/w) of the pineapple weight^38^. These byproducts are inexpensive raw materials used for high-value-added product formation. Previous studies have reported that the byproducts, particularly pineapple peels and cores (PPC), possess high contents of numerous biodegradable substances, including phenolic compounds, vitamins, minerals, proteolytic enzymes, sugars, and crude fibers, that provide beneficial antioxidants, antimicrobial and anti-inflammatory health effects^39–41^. Thus, PPC is considered a promising nutritional material for increasing the valuable components of functional products. Several studies have provided clues on enhancing the health-promoting properties of kombucha supplementation with various nutritional ingredients; however, using fresh PPC as a valuable enrichment of kombucha tea still requires better documentation. Therefore, this research is designed to evaluate PPC supplements combined with black tea to alter the chemical compositions and promote the health benefits of kombucha products.

## Materials and methods

### Plant material

Pineapple (*Ananas comosus* L. cv. Pattavia) was collected from the Office of Agricultural Research and Development (ARD) Region 2, Thailand, with the permission of the ARD office. Dried black tea leaves were purchased from the Royal Project Tea Highland, Chiang Mai, Thailand. Neither pineapple nor black tea are wild but are cultivated in Nong Khai and Chiang Mai provinces, respectively. The sample collection and preparation methods followed relevant guidelines and legislation.

### Preparation of raw materials and chemical composition analyses

The pineapple fruits were washed thoroughly with running tap water to remove dirt and soil particles. Then, the PPC was collected after peeling off the washed pineapple fruit, and the resulting PPC was cut into small pieces (approximately 0.5 × 0.5 cm). The fresh-cut PPC was ground using a blender (HR2225/00, Philips, China) and kept at − 20 °C until it was used. For preparation of sweetened black tea, 4.5 g of dried black tea leaves was steeped in 1 L of hot water for 10 min. After removing the tea leaves by filtration through four layers of cheesecloth, 60 g of brown sugar (Mitr Phol Sugar Corp., Ltd., Thailand) was added and mixed until the sugar was completely dissolved. The resulting sweetened tea was used as the starting material for further experiments. The chemical compositions of the fresh PPC and dried black tea, such as the total sugar (TS) content, TPC, TFC, and antioxidant activity, were determined.

### Preparation of kombucha SCOBY

The kombucha starter culture, or kombucha SCOBY (Chiira Organic, Thailand), was prepared using the modified method of Torán-Pereg et al.^42^ Briefly, 10% (v/v) kombucha SCOBY was added into sterile glass jars containing 900 mL of sweetened tea, and the mouths of the jars were covered with clean cheesecloth and incubated at room temperature in the dark. After 14 days of incubation, the resulting kombucha SCOBY was used as a starter culture for kombucha production in subsequent experiments.

### Determination of the microbial community in the kombucha SCOBY

The microbial community of the kombucha SCOBY was determined within 3 and 7 days after fermentation in sweetened black tea. The total genomic DNA (gDNA) of bacteria and yeasts in the kombucha SCOBY was isolated using a procedure described by Kanwal et al.^43^ with minor modifications. The purity and concentration of the gDNA were assessed using a BioDrop μLite (Denville Scientific Inc., New Jersey, US), according to the manufacturer’s instructions, and the quality of the DNA was monitored with a 1% agarose gel (Smart Agarose-S BIO, Spain). The purified gDNA samples were shipped to Novogene Co., Ltd., Beijing, China, for PCR amplification, library preparation, and sequencing. The conserved primers 341F (5'-CCTAYGGGRBGCASCA-3') and 806R (5'-GGACTACNNGGGTATCTAA-3') were used to amplify the V3–V4 region of the bacterial 16S rRNA gene for bacterial identification, and primers ITS5-1737F (5'-GGAAGTAAAAGTCGTAACAAGG-3') and ITS2-2043R (5'-GCTGCGTTCTTCATCGATGC-3') were used to amplify the internal transcribed spacer (ITS) region for fungal identification. High-throughput sequencing was performed using the Illumina NovaSeq PE250 platform (Novogene Co., Ltd., Beijing, China), and 250-bp paired-end reads were generated and analyzed. Briefly, targeted regions were amplified using specific primers connecting with barcodes, and the PCR products with proper size were selected by 2% agarose gel electrophoresis. The exact amount of PCR products from each sample were pooled, end-repaired, A-tailed, and ligated with Illumina adapters. After the quantification and size distribution analysis, the quantified libraries were pooled and sequenced on the Illumina platform to generate 250 bp paired-end raw reads according to a standard Illumina protocol. After the barcode and primer sequences were truncated, the paired-end reads were merged using FLASH (Version 1.2.11; http://ccb.jhu.edu/software/FLASH/)^44^, and the raw tags were obtained. Quality filtering on the raw tags was performed using the FASTP (Version 0.20.0) software to get high-quality clean tags. A Vsearch (Version 2.15.0) software was used to blast clean tags with the reference database (Silva database for 16S/18S; https://www.arbsilva.de/, and Unite database for ITS; https://unite.ut.ee/) to detect the chimera sequences^45^. After removing chimera sequences, the resulting effective tags were denoised using the Divisive Amplicon Denoising Algorithm 2 (DADA2) or deblur module in the Quantitative Insights into Microbial Ecology (QIIME2) software (Version QIIME2-202,006), and the sequences with less than 5 abundance (n < 5 within all samples) were filtered out to obtain final amplicon sequence variants (ASVs). The taxonomic classification of the phylum, class, order, family, genus, and species was performed using the Classify-sklearn moduler in QIIME2 software based on operational taxonomic units (OTU) annotation^46^.

### Production of kombucha beverage from a fusion of black tea and PPC

**Figure 1:**
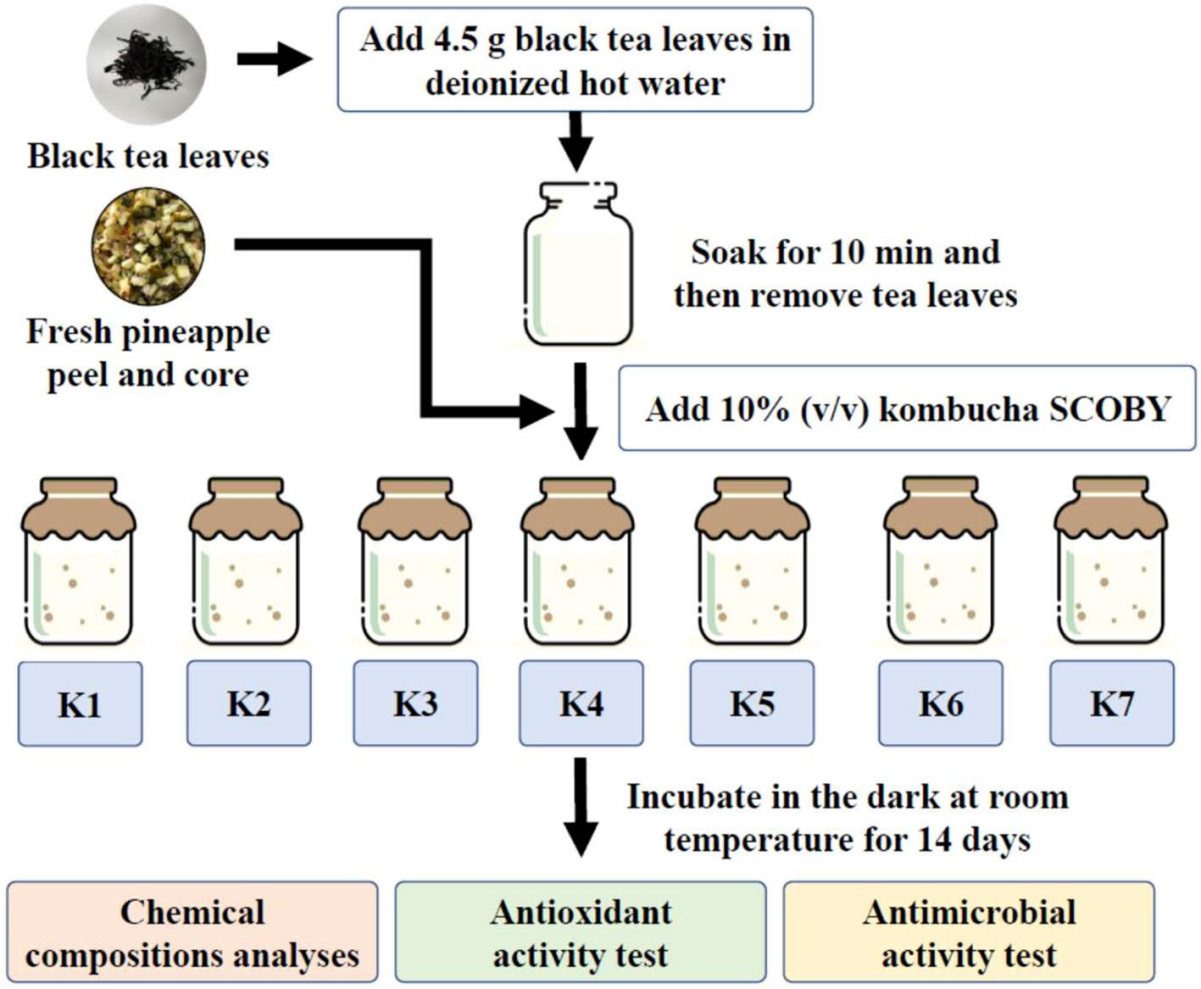
A process flow diagram for kombucha fermentation experiments. (K1: 60 g of brown sugar and 0 g of PPC; K2: 50 g of brown sugar and 10 g of PPC; K3: 40 g of brown sugar and 20 g of PPC; K4: 30 g of brown sugar and 30 g of PPC; K5: 20 g of brown sugar and 40 g of PPC; K6: 10 g of brown sugar and 50 g of PPC; and K7: 0 g of brown sugar and 60 g of PPC).

Kombucha beverages were produced from a fusion of black tea and PPC using the procedure described by Jayabalan et al.^1^, Kaewkod et al.^6^, and Jakubczyk et al.^7^, with slight modifications. Briefly, 4.5 g of dried black tea leaves was steeped in 1 L of deionized hot water for 10 min. After removing the tea leaves by filtration through four layers of cheesecloth, the resulting hot tea was used as a fermentation medium for kombucha production. Seven formulations of the kombucha beverages using different sugar contents in the brown sugar and PPC were produced, i.e., K1: 60 g of brown sugar and 0 g of PPC; K2: 50 g of brown sugar and 10 g of PPC; K3: 40 g of brown sugar and 20 g of PPC; K4: 30 g of brown sugar and 30 g of PPC; K5: 20 g of brown sugar and 40 g of PPC; K6: 10 g of brown sugar and 50 g of PPC; and K7: 0 g of brown sugar and 60 g of PPC. The total sugar concentrations in the fermentation mixtures were adjusted to 60 g/L with pineapple juice extracted from the PPC to ensure the validity of the kombucha production comparisons. A kombucha starter culture was added to the fermentation mixtures to give a final concentration of 10% (v/v). The mouths of the fermentation jars were carefully covered with four layers of cheesecloth, and the jars were placed in the dark at room temperature to allow fermentation for 14 days. During the fermentation, kombucha samples were withdrawn every two days, and the chemical profiles and biological properties, including antioxidant and antimicrobial activities, were analyzed. Kombucha K1 without PPC supplementation was used as a control treatment in this study. Figure 1 shows a process flow diagram of kombucha fermentation experiments. Three independent replicates were carried out for each kombucha production formulation, and the results are presented as the mean ± standard deviation (SD).

### Antioxidant activity assay

#### Antioxidant activity using ferric ion reducing power (FRAP)

The total reduction potential or antioxidant activity of a kombucha sample was determined from its ability to reduce Fe^3+^ to Fe^2+^ by using the ferric ion reducing power (FRAP) method described by Amanzadeh-Salout et al.^47^ with some modifications. The FRAP reagent was prepared by mixing 100 mL of acetate buffer, 10 mL of 10 mM 2,4,6-Tris(2-pyridyl)-s-triazine (TPTZ) solution, and 10 mL of 20 mM FeCl_3_.3H_2_O solution. Fifty microliters of the kombucha sample was mixed with 1.5 mL of the FRAP reagent and 150 μL of distilled water, and the reaction mixture was kept in the dark at room temperature for 10 min. After incubation, the absorbance of the reaction mixture was measured at a wavelength of 593 nm. The FRAP antioxidant activity was determined from a calibration curve generated with ferrous sulfate heptahydrate as the reference standard and expressed in millimolar Fe(II) per liter of kombucha (mM Fe(II)/L).

#### Antioxidant activity using the 2,2’-azinobis-(3-ethylbenzothiazoline-6-sulfonate) (ABTS) radical scavenging assay

The scavenging activities of the radical ABTS^+^ were determined based on the modified method described by Mukherjee et al.^48^ For the preparation of the ABTS•^+^ reagent, an ABTS stock solution (7 mM) was mixed with 2.45 mM potassium persulfate and incubated overnight in the dark at room temperature. The ABTS•^+^ stock reagent was diluted with distilled water to give an absorbance of 0.700 at 734 nm, and the resulting dilution ratio was used to prepare the reaction solution. A hundred microliters of the diluted sample solution was mixed with a 3.8 mL reaction solution. After 6 min of incubation in the dark at room temperature, the absorbance of the reaction mixture was measured at a wavelength of 734 nm. Ascorbic acid was used as a standard reference, and the percentage of inhibition by the ABTS^+^ was calculated using the following equation: % inhibition = [(A_control_ − A_sample_)/(A_control_)] × 100, where A_control_ is the diluted ABTS•^+^ reagent without sample solution, and A_sample_ is the reaction mixture of the sample solution and diluted ABTS•^+^ reagent.

### Antimicrobial activity assay

The antimicrobial activity of kombucha against pathogenic bacteria was tested with the agar well diffusion method using the protocol described by Kaewkod et al.^6^ with slight modifications. The bacterial strains used in this study, including gram-negative bacteria (*Aeromonas hydrophila, Escherichia coli* ATCC 25922, *Pseudomonas aeruginosa* ATCC 27853, and *Salmonella typhi* DMST 22842) and gram-positive bacteria (*Bacillus cereus*) were kindly obtained from the Faculty of Associated Medical Science, Khon Kaen University, Thailand. For the antimicrobial activity test, 200 μl of bacterial culture was transferred into brain heart infusion (BHI) broth medium and incubated at 37 °C and 150 rpm for 18–24 h. The growth of the bacterial culture was measured by spectrophotometry at 600 nm, and it was swabbed on Mueller–Hinton (MH) agar (Himedia ™, India). Wells 6 mm in diameter were prepared on agar plates with a sterile cork borer. The kombucha samples were centrifuged at 10,000 rpm for 5 min to remove the cell debris, and the supernatant was then filtered through a 0.22 μm sterile microfilter and transferred into the agar wells. The plates were kept at 4 °C for 2 h and then incubated at 37 °C for 18–24 h. The inhibition zones for bacterial growth were determined as described by Battikh et al.^8^ A comparison of the antimicrobial activity of fresh kombucha was conducted with acetic acid as a positive control and distilled water as a negative control. For control treatment preparation, 8.5 g/L acetic acid was mixed with sterile distilled water, and the pH was adjusted to 3.0 (the same pH as the kombucha samples). The resulting solution was sterilized by filtration and used to test antimicrobial activity along with the kombucha samples.

### Analytical methods

The chemical composition of the PPC, including the sugars (such as glucose, fructose, and sucrose), vitamins (B1, B2, B3, B6, B9, and B12), and organic acids (citric acid, ascorbic acid, and formic acid), were analyzed by using high-performance liquid chromatography (HPLC) with a refractive index (RI) detector (Shimadzu, Kyoto, Japan) and an Aminex HPX-87H column (300 × 7.8 mm) (Bio-Rad, Hercules, CA, USA) at 50 °C. Mineral contents (nitrogen, phosphorus, potassium, magnesium, manganese, zinc, ferrous, copper, calcium, sodium, cobalt, and molybdenum) were determined by atomic absorption spectroscopy (AAS) at the Central Laboratory (Thailand) Co., Ltd., Khon Kaen branch, Thailand. The pHs of the kombucha samples were measured with an electronic pH meter (FE28 FiveEasy, Mettler Toledo, Switzerland), and total acidity was determined using the method described by Osiripun et al.^33^ The color of the kombucha products was determined on a scale L*, a* and b* with a standard reference method using Ultrascan XE SN-U3115 (Hunterlab, Reston, Virginia, USA). TS in the kombucha was analyzed with the phenol–sulfuric acid method.^49^ TPC was determined by the Folin–Ciocalteu method^7^ using gallic acid as a standard, and the results were expressed as gram gallic acid equivalents (GAE) per liter of the kombucha beverage (g GAE/L). TFC was determined by the aluminum chloride colorimetric assay^50^ using quercetin as a standard. The TFC was expressed as milligrams of quercetin equivalent (QE) per liter of the kombucha beverage (mg QE/L). The ethanol concentration in the kombucha was determined by gas chromatography (GC) (GC-14B, Shimadzu, Kyoto, Japan) using a polyethylene glycol (PEG-20 M)-packed column with a flame ionization detector (FID). Volatile compounds in the kombucha beverages were determined using a GC (Agilent 7890A) equipped with a mass spectrometer (Agilent 7000B, Agilent Technologies, Inc., Palo Alto, CA, USA). Analyte separation was assessed with a DB-wax capillary column (60 m long, 0.25 mm in diameter, and 0.25 μm in film thickness).

### Statistical analyses

A complete randomized design (CRD) was used for the kombucha fermentation experiments with three replications, and all data were expressed as the mean ± SD. The significant differences among groups were evaluated using SPSS version 28.0. (IBM SPSS Statistics, Armonk, NY: IBM Corp.) A statistically significant level was defined with the one-way analysis of variance (ANOVA) followed by an LSD post hoc test at a level of 0.05 (*p* ≤ 0.05 means significance).

## Results and discussion

### Chemical compositions of the raw materials

The chemical compositions of the black tea and fresh PPC, including TS, TPC, TFC, and antioxidant activity, were assessed, and the results are summarized in Table 1. The TS and TPC of the PPC were approximately 315- and twofold higher than those of black tea. However, black tea exhibited significantly higher TFC and antioxidant activity than PPC. Based on the FRAP and ABTS assays, black tea exhibited strong free radical inhibition capacity (0.82 mM Fe(II)/L) and radical scavenging ability (89.94% inhibition), approximately 1.7- and 1.5-fold, respectively, greater than those of PPC. The high antioxidant activity of black tea may be associated with the high levels of polyphenols and flavonoids, as in a study by Zhang et al.^51^, who reported a higher phenolic content and more vital antioxidant ability in black tea. Due to a low level of TS (0.15 g/L), black tea is a non-sweetened or low-calorie beverage. PPC exhibited a lower pH than black tea, which may correlate with a high acidity level, specifically citric acid and ascorbic acid. The pH of the black tea was 4.60, meaning that black tea is mildly acidic due to the acidity of the tea leaves. One of the dominant acids in black tea is tannic acid (accounting for 13.36%), which plays a crucial role in determining the black tea flavor^52,53^. Previous studies demonstrated that acidic conditions (pH less than 7.0) maintained the stabilities of the beneficial compounds in the raw material^54^. Hence, black tea and PPC are potential sources of phenolic and flavonoid compounds that could play vital roles in the strong antioxidant capacities of kombucha products.

**Table 1:** Sugar, phenolic, flavonoid contents, and antioxidant activities of black tea and fresh pineapple peels and cores (PPC).

Parameters	Black tea	Fresh PPC
Total sugar (g/L)	0.15 ± 0.01	47.25 ± 3.40
Total phenolic content (g GAE/L)	0.05 ± 0.00	0.10 ± 0.02
Total flavonoid content (mg QE/L)	6.54 ± 1.25	2.97 ± 0.41
FRAP (mM Fe(II)/L)	0.82 ± 0.05	0.49 ± 0.06
ABTS (% inhibition)	89.94 ± 0.84	58.97 ± 1.93
pH value	4.60 ± 0.02	3.21 ± 0.01
Moisture (%)	ND	83.25 ± 1.20

*ND* not determined.

Results are presented as mean ± SD of three independent experiments.

Naturally, phenolics and flavonoids mainly contribute to plant antioxidant properties, which enhance free radical inhibition due to the capacity of the hydroxyl group to donate a hydrogen atom to a free radical^55^. In this study, the TPC and TFC of PPC accounted for 0.10 g of GAE/L and 2.97 mg of QE/L, respectively, and the radical inhibition capacity and scavenging activity of PPC were 0.49 mM Fe(II)/L and 58.97%, respectively, which were similar to those reported by Azizan et al.^39^ Regarding the sugar content, glucose and fructose were the significant sugars detected in PPC, accounting for 22.93 and 18.87 g/kg of fresh weight (FW). The sucrose content accounted for 10.80 g/kg FW (Table 2). Based on these findings, PPC could be used as an alternative source of sugar to partially or entirely replace sucrose, one of the most commonly used sugars for kombucha production.

**Table 2:** Chemical compositions of fresh PPC.

Characterization	Nutrients	Fresh PPC
Sugars (g/100 g)	Glucose	22.93
	Fructose	18.87
	Sucrose	10.80
Organic acids (mg/100 g)	Citric acid	590.00
	Ascorbic acid	0.02
	Formic acid	ND
Vitamins (mg/100 g)	B1 (Thiamine)	0.05
	B2 (Riboflavin)	ND
	B3 (Niacinamide)	0.07
	B6 (Pyridoxine)	0.01
	B9 (Folic acid)	0.01
	B12 (Cyanocobalamin)	ND
Minerals (mg/kg)	Nitrogen (N)	1410.00
	Phosphorus (P)	63.78
	Potassium (K)	991.95
	Magnesium (Mg)	77.80
	Calcium (Ca)	72.30
	Zinc (Zn)	71.75
	Manganese (Mn)	31.24
	Iron (Fe)	7.60
	Sodium (Na)	16.96
	Copper (Cu)	20.85

Citric acid was the predominant organic acid detected in the PPC, accounting for 590 mg/100 g FW. It should be noted that the ascorbic acid or vitamin C contents in the PPC constituted 0.02 mg/100 g FW, which was markedly lower than that reported by Mohsin et al.^40^ (47.90 mg/100 mg FW). This finding might be attributable to the differences in plant species, harvesting stages, and processing methods of the pineapple fruit. Surprisingly, PPC contains many vitamins, mainly belonging to Group B, which refers to the water-soluble vitamin group (the B1, B3, B6, and B9 contents were 0.05, 0.07, 0.01, and 0.01 mg/100 g FW, respectively). Vitamins B2 and B12 were not detected in this study. All of the detected vitamins in PPC are known to benefit human health, e.g., B1 is an essential cofactor for many enzyme-catalyzed reactions in different carboxylation metabolisms, while B3 is a cofactor for many enzymes that participate in various metabolic pathways and lower the level of cholesterol and improve blood circulation. B6 is essential for converting homocysteine to methionine, which lowers the homocysteine level in the body. Furthermore, B9 is a cofactor in the enzyme-catalyzed transfers of carbon atoms in many metabolic pathways, including the biosyntheses of purines and pyrimidines (essential for DNA and RNA) and the interconversions of amino acids^56,57^. In the case of pineapple nutrition, several studies have reported that B1 (0.05–0.14 mg/100 g), B3 (0.13–0.27 mg/100 g), and B6 (0.08–0.11 mg/100 g) are the most predominant among all vitamins^58^.

In addition to sugars, organic acids, and vitamins, PPC also contains several minerals or elements, some of which are essential for microbial growth and metabolic activity. Nitrogen (N) was the main element detected in the PPC, accounting for 1410 mg/kg FW, followed by potassium (K) (991.95 mg/kg), magnesium (Mg) (77.80 mg/kg), calcium (Ca) (72.30 mg/kg), zinc (Zn) (71.75 mg/kg), and the lowest was iron (Fe) (7.60 mg/kg) (Table 2). N and phosphorus (P) are essential for protein and nucleic acid biosyntheses, while Fe, Mg, and Zn are necessary for microbial growth and serve as cofactors for several enzymes in the microbial biochemical reaction pathway^59^. K is required for the normal functioning of all cells, and it regulates the heartbeat, ensures the activities of muscles and nerves, and supports protein synthesis and carbohydrate metabolism. It was noteworthy that the contents of K and Zn in the PPC used in this study were significantly higher, whereas those of Mg, Ca, and manganese (Mn) were considerably lower than those reported by Lu et al.^60^ and Romelle et al.^61^, which might be due to the different plant varieties, growing environmental conditions, and harvesting stages.

### Microbial community in kombucha SCOBY

**Figure 2:**
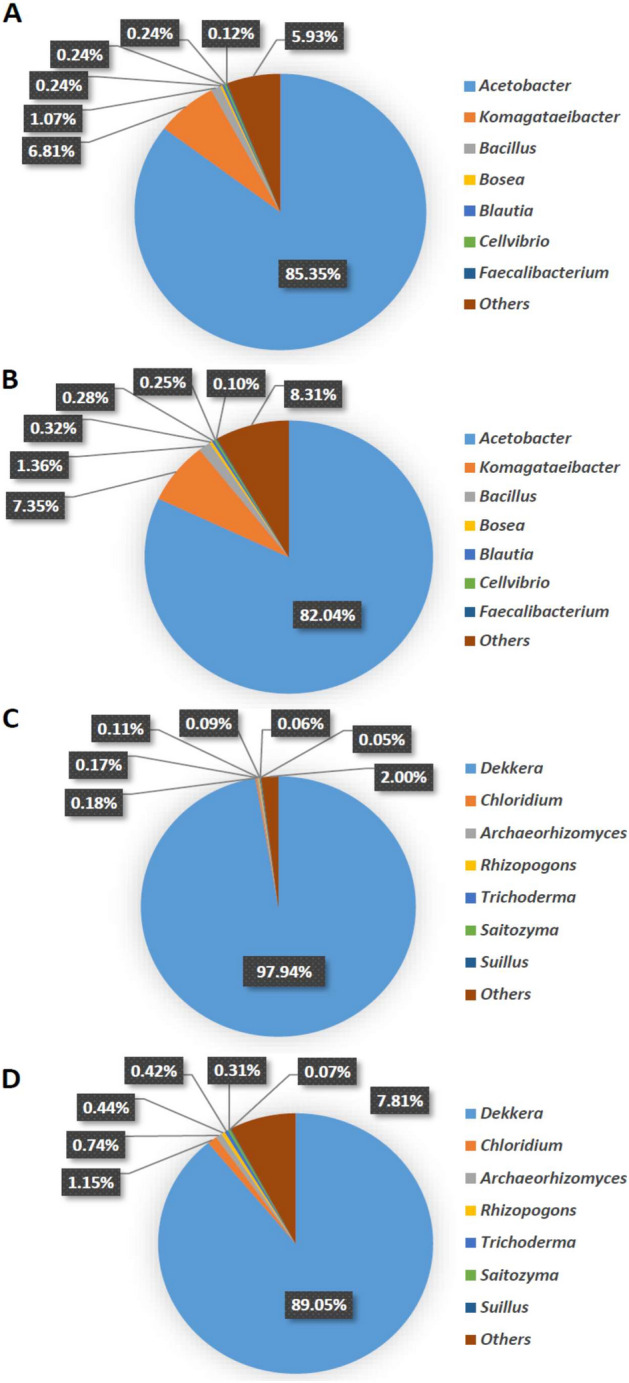
Microbial community in kombucha SCOBY. Bacterial diversity in kombucha SCOBY after 3 days (**A**) and 7 days (**B**) of fermentation. Fungal diversity in kombucha SCOBY after 3 days (**C**) and 7 days (**D**) of fermentation.

Microorganisms found in the kombucha SCOBY were mainly acetic acid bacteria (AAB) and yeasts, which significantly impact the biochemical properties of the kombucha products, and the diversity of microbes may vary depending on the source of SCOBY, cultivation medium, fermentation conditions, and the raw materials used for kombucha production^1,5^. Figure 2 illustrates the microbial diversity of 3- and 7-day kombucha SCOBY based on next-generation sequencing analyses. Several bacteria were identified, such as *Acetobacter*, *Komagataeibacter*, *Bacillus*, *Bosea*, *Blautia*, *Cellvibrio*, and *Faecalibacterium*. Among the bacteria found in the 3-day kombucha SCOBY, the most predominant were *Acetobacter* and *Komagataeibacter,* accounting for 85.35 and 6.81% of the total, respectively (Fig. 2A). These two bacteria are classified as AAB, which are responsible for the production of organic acids, such as acetic acid, glucuronic acid, gluconic acid, ascorbic acid, and succinic acid. Furthermore, they also form cellulose biofilms and promising detoxifying and antioxidant compounds, specifically DSL^1,11,62^. Several studies have also noted that AAB were found in kombucha products at higher levels than lactic acid bacteria (LAB)^63,64^. A recent study by Lee et al.^65^ demonstrated that the main AAB found in several commercial kombucha SCOBY samples were *Gluconobacter* (95.25%) and *Komagataeibacter* (76.69%). The predominant LAB detected in kombucha primarily belonged to the *Lactobacillus* group, which enhances the biological function as probiotic kombucha beverages^66,67^. However, LAB were not detected in the present study. *Bacillus* is another bacterium found in kombucha SCOBY, accounting for 1.07%, which is responsible for lactic acid production. *Bacillus* species have also been utilized as probiotics in several food and beverage products. In food fermentation, some *Bacillus* probiotics are used as the key factor in fermenting soybean and dietary products to generate various antioxidants and antimicrobials^68^. *Bacillus* strains combined with plant secondary metabolites enhanced the apparent bioavailability and digestibility in the intestinal system^69^. In addition to AAB and *Bacillus*, other bacteria present in the kombucha SCOBY, such as *Blautia* sp. and *Faecalibacterium* sp., may also play roles during kombucha fermentation, even though they were found in a relatively low number. Based on the literature reviews, *Blautia* sp. and  *Faecalibacterium* sp. are known as producers of acetic acid, butyric acid, and short-chain fatty acids. Furthermore, both strains are considered to be potential next-generation probiotics that may play a role in regulating host health and alleviating metabolic syndromes^70,71^.

During kombucha fermentation, the microbial community exhibited a noticeable change in the bacterial diversity, mainly in the 7-day kombucha SCOBY (Fig. 2B). The percentage of *Acetobacter* decreased slightly from 85.35 to 82.04%, while the proportions of *Komagataeibacter* and *Bacillus* increased to 7.35 and 1.36%, respectively, which might have been due to the biochemical changes in kombucha that affect bacterial growth and development. Previous studies reported that *Acetobacter* participated in ethanol oxidation and was strongly associated with a highly alcoholic environment, while *Komagataeibacter* utilizes glucose, fructose, sucrose, and ethanol during metabolism^72,73^.

Yeasts also play vital roles in the hydrolysis of sucrose into glucose and fructose and finally to ethanol, which influences the flavors and aromas of kombucha products. In this study, *Dekkera* was the predominant yeast in the 3-day kombucha SCOBY, accounting for 97.94% (Fig. 2C). The falling pH value of the kombucha during fermentation caused a reduction in the growth of *Dekkera*, which can be seen from the 7-day kombucha SCOBY; the percentage of *Dekkera* had decreased from 97.94% to 89.05% (Fig. 2D). *Dekkera* produces high levels of ethanol and is associated with the production of organic acids and esters, which influence the kombucha aroma and make it a good choice for alcoholic beverages and bioethanol production^73–75^. Tran et al.^76^ reported that *Dekkera* exhibited relatively high invertase activity and fermentative efficiency in producing high ethanol contents.

In addition to *Dekkera* sp., several other yeasts were also detected in relatively low abundance, such as *Chloridium*, *Archaeorhizomyces*, *Trichoderma*, *Rhizospogon*, *Saitozyma,* and *Suillus*, most of which are plant-associated fungi found on plant surfaces and in the rhizosphere. Although some are known to produce cellulolytic enzymes, such as *Trichoderma*, others, such as *Saitozyma*, are known to produce gluconic acid^77,78^. The small presence of these fungi may not play any role in the kombucha process. Notably, the common ethanologenic yeast *Saccharomyces cerevisiae*, a well-known ethanol producer in alcoholic beverages, was not detected in this study, which might be attributable to the different sources of kombucha SCOBY and fermentation conditions.

### Kombucha production and chemical compositions of the kombucha products

**Figure 3:**
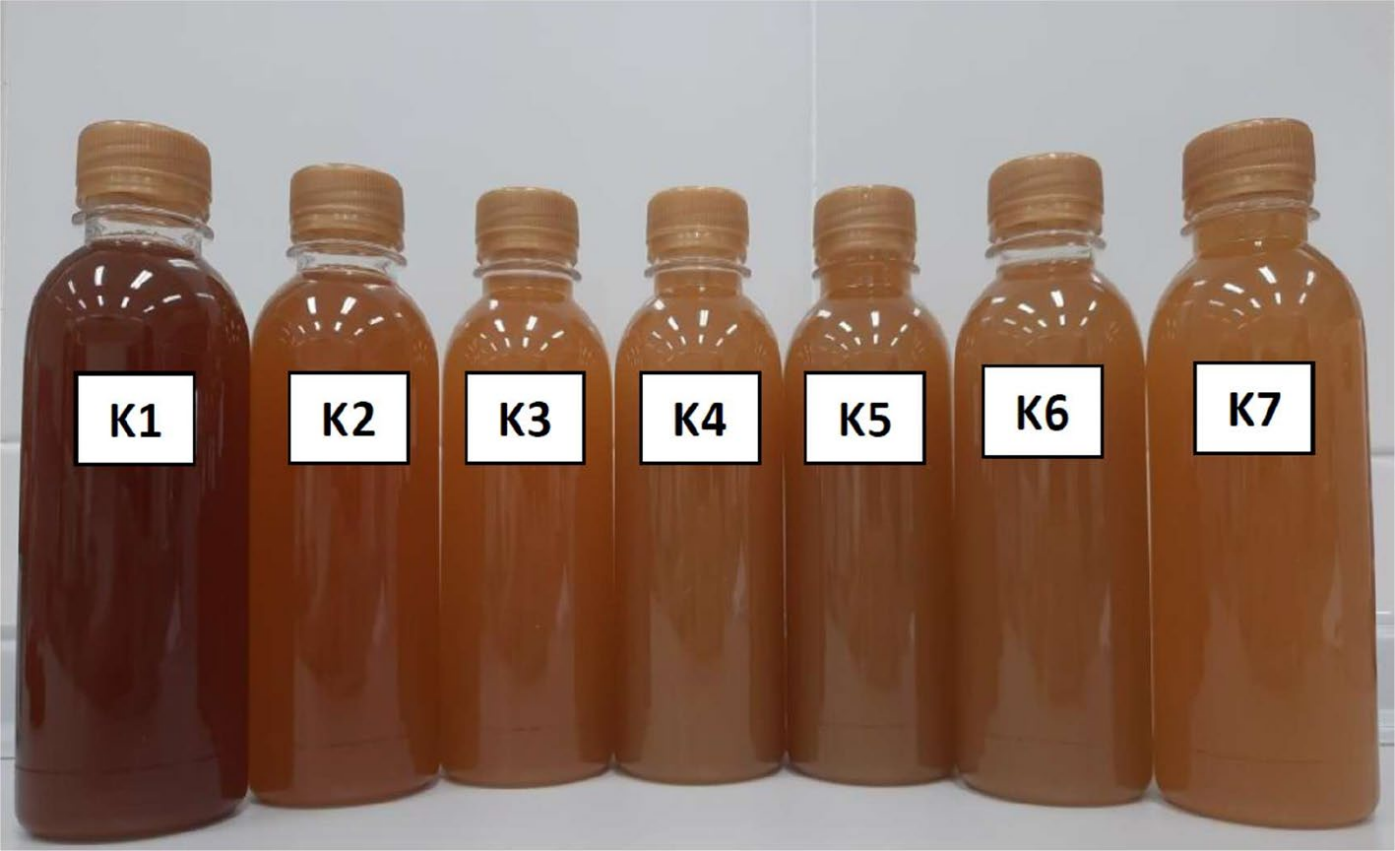
The appearance of kombucha products made from black tea and fusions of black tea and PPC using different sugar contents in the brown sugar and PPC as described in “Materials and Methods”. (K1: 60 g of brown sugar and 0 g of PPC; K2: 50 g of brown sugar and 10 g of PPC; K3: 40 g of brown sugar and 20 g of PPC; K4: 30 g of brown sugar and 30 g of PPC; K5: 20 g of brown sugar and 40 g of PPC; K6: 10 g of brown sugar and 50 g of PPC; and K7: 0 g of brown sugar and 60 g of PPC).

During the fermentation of kombucha, a biofilm, identified as the cellulose layer to which microbial cells are attached, was visible within 3 days and floated on top of the culture medium within 14 days of fermentation. The color of the kombucha products varied depending on the types and ratios of the raw material used. As shown in Table 3, the L* values of kombucha produced from fusions of black tea and PPC ranged from 88.68 to 90.85, which were higher than that of the control kombucha (74.60), indicating that the kombucha products from fusions of black tea and PPC were clearer than the control kombucha. On the other hand, a* and b* values of the control kombucha were higher than kombucha products from fusions of black tea and PPC, indicating a more red-yellowish color than those produced from fusions of black tea and PPC (Fig. 3).

**Table 3:** Color values of kombucha products made from black tea and fusions of black tea and PPC.

Sample	L*	a*	b*
K1	74.60 ± 0.01	11.41 ± 0.01	58.72 ± 0.01
K2	89.36 ± 0.02	0.88 ± 0.01	38.45 ± 0.04
K3	90.85 ± 0.04	0.23 ± 0.03	34.48 ± 0.04
K4	89.74 ± 0.01	0.79 ± 0.01	32.45 ± 0.04
K5	88.68 ± 0.02	1.25 ± 0.02	33.50 ± 0.01
K6	89.58 ± 0.03	0.80 ± 0.01	30.70 ± 0.03
K7	89.97 ± 0.06	0.73 ± 0.01	29.56 ± 0.08

**Figure 4:**
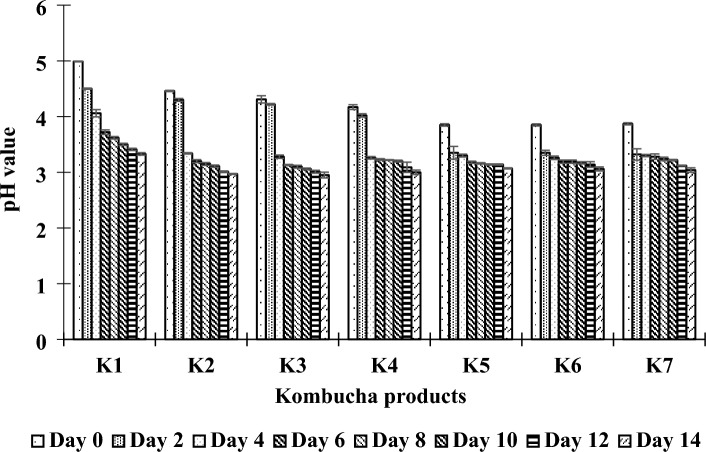
Changes in pH values during fermentation of kombucha products made from black tea and fusions of black tea and PPC using different sugar contents in the brown sugar and PPC as described in “Materials and Methods”.

The initial pH of the control kombucha (K1) (pH 4.99) was slightly higher than those of kombuchas supplemented with PPC (K2–K7), which ranged from 3.85 to 4.46. Increasing the PPC supplementation decreased the kombucha pH values, which might be correlated with the high acidity since the PPC contains a high organic acid content, specifically due to citric acid and ascorbic acid (Table 2). During fermentation, the pH of the kombucha dramatically dropped from 3.85–4.99 to 2.95–3.30, which was in the acceptable range for kombucha beverages based on the recommendation of the Food and Drug Administration (FDA)^5^. Notably, the decreases in pH for the kombuchas produced from fusions of black tea and PPC (K2-K7) were more significant than that of the control kombucha (K1) (Fig. 4). The decrease in pH over the fermentation period indicated that the bacteria and yeasts in the kombucha SCOBY converted sugars into various organic acids, such as acetic acid, gluconic acid, glucuronic acid, lactic acid, and other secondary metabolites^79^. Furthermore, a low kombucha pH also limits the undesirable growth of microbial contaminants^4^. It should be noted that the final pH values of the kombucha products may be correlated with the different substances released from the raw materials or generated during fermentation, which may affect the microbial growth and metabolic activities of the kombucha SCOBY^79^.

**Figure 5:**
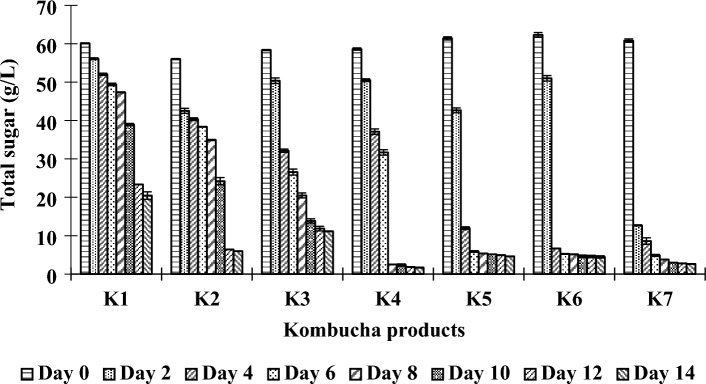
Changes in total sugar contents during fermentation of kombucha products made from black tea and fusions of black tea and PPC using different sugar contents in the brown sugar and PPC as described in “Materials and Methods”.

The initial sugar concentrations of the kombucha beverages were approximately 60 g/L. Changes in the TS contents were observed during the fermentation process due to the microbial activities of the kombucha SCOBY, which utilized sugar as a carbon source to grow and produce beneficial bioactive substances. There were dramatic reductions in the sugar contents of all kombucha products, especially during the first 2 to 8 days of fermentation, depending on the ratios of raw materials used. A high proportion of PPC gave a high rate of sugar consumption, which may be attributable to glucose and fructose, the major sugars found in PPC, which are more efficiently taken up by microbial cells than other sugars. The final TS concentrations of the kombuchas produced from fusions of black tea and PPC (K2–K7) ranged from 1.70 to 11.13 g/L, which were lower than that of the control kombucha (20.45 g/L) (K1) (Fig. 5). This finding suggested that the PPC enhanced the growth and metabolic activities of the microbial community in the kombucha SCOBY that utilized the sugars as nutrient sources supporting the production of valuable metabolites. It should be noted that the final TS concentrations in the kombucha products of the current study were lower than those reported by Osiripun et al.^33^ and Lee et al.^65^, which might be due to the differences in initial TS contents, the diversity and metabolic activities of the microbial communities of the kombucha SCOBY, nutritional supplementation levels, and the fermentation conditions.

Kombucha is known to contain high amounts of polyphenols, which come from tea leaves and are also generated during fermentation by the microbial community in the kombucha SCOBY. Compared with the raw materials, the kombucha products made from fusions of black tea and PPC displayed higher phenolic contents than the original black tea and PPC materials. The phenolic contents of the kombucha products tended to increase at the end of fermentation, specifically for those supplemented with 30–60 g/L PPC, compared to those supplemented with 10–20 g/L PPC and the control treatment without PPC addition. The increased phenolic contents of the kombucha products made from fusions of black tea and PPC may be correlated with the release of polyphenols from the PPC, as seen in other studies using wheatgrass juice^26^, black carrots^80^, and medicinal plants^81^. Previous studies have demonstrated that PPC contains various beneficial chemical compounds, and polyphenols are among those compounds^61,82,83^.

The final phenolic content of the control kombucha was 0.28 g GAE/L, while kombuchas made from fusions of black tea and PPC had phenolic contents in the range of 0.27–0.34 g GAE/L (Table 4). The fluctuations seen in the phenolic content during fermentation may be associated with the metabolic activity of the microbial community in the kombucha SCOBY. The synergistic effect of enzymes, such as cellulase, glucanase, pectinase, and glucosidase, produced by the bacteria and yeast in the kombucha SCOBY, can degrade the polyphenol complexes in the raw materials into smaller polyphenol monomers, increasing the phenolic contents of the kombucha products. Another possibility is that the plant cell walls, such as those of the tea, contained high levels of insoluble bound phenols, which were difficult to release into the kombucha products. The beneficial metabolites produced by the microbial community during kombucha fermentation, such as acids, alcohols, and esters, can affect the solubility of the phenolic compounds, leading to the liberation of insoluble bound phenols^67^. As per the reduction in TPC seen in the kombucha products, the microbial community in the kombucha SCOBY can also release hydrolytic enzymes to degrade the polyphenols into other metabolites and use them as nutrient sources for metabolic activities, leading to a reduction in the TPC^6,84,85^. Polymerization of the polyphenols into high molecular weight complexes may also inhibit the detection of these compounds in the kombucha products^86^; thus, depolymerization of the polymerized active compounds might increase the measured total phenol content.

**Table 4:** Total phenolic contents of kombucha products made from black tea and fusions of black tea and PPC.

Time (Day)	Kombucha products						
	K1	K2	K3	K4	K5	K6	K7
0	0.28 ± 0.00^BCb^	0.28 ± 0.00^Aab^	0.28 ± 0.00^ABb^	0.29 ± 0.00^ Da^	0.26 ± 0.00^Fc^	0.25 ± 0.00^Fcd^	0.25 ± 0.00^Hd^
2	0.29 ± 0.00^ABbc^	0.28 ± 0.00^Acd^	0.29 ± 0.00^Ab^	0.30 ± 0.01^ABa^	0.28 ± 0.01^Ed^	0.30 ± 0.00^ Da^	0.28 ± 0.00^Gd^
4	0.29 ± 0.00^ABbc^	0.28 ± 0.00^Bde^	0.27 ± 0.00^Ce^	0.28 ± 0.00^Ecd^	0.30 ± 0.00^ Da^	0.30 ± 0.00^Ea^	0.29 ± 0.01^Fb^
6	0.29 ± 0.00^Ad^	0.25 ± 0.00^Fg^	0.26 ± 0.00^Df^	0.27 ± 0.00^Fe^	0.30 ± 0.00^Cc^	0.32 ± 0.00^Cb^	0.34 ± 0.00^Ba^
8	0.28 ± 0.00^Ce^	0.26 ± 0.00^Ef^	0.27 ± 0.00^BCe^	0.29 ± 0.00^Dd^	0.30 ± 0.00^CDc^	0.32 ± 0.00^Cb^	0.34 ± 0.00^Aa^
10	0.28 ± 0.01^Cd^	0.26 ± 0.01^De^	0.28 ± 0.00^ABd^	0.29 ± 0.00^Cc^	0.31 ± 0.00^Cb^	0.33 ± 0.00^Ba^	0.31 ± 0.00^Db^
12	0.28 ± 0.00^Ce^	0.27 ± 0.00^Cf^	0.29 ± 0.00^Ad^	0.31 ± 0.01^Ab^	0.31 ± 0.00^Bb^	0.33 ± 0.00^Aa^	0.30 ± 0.00^Ec^
14	0.28 ± 0.00^BCd^	0.27 ± 0.00^BCe^	0.29 ± 0.00^Ad^	0.31 ± 0.01^Ac^	0.32 ± 0.00^Ab^	0.34 ± 0.00^Aa^	0.33 ± 0.00^Cb^

Data are mean ± SD (expressed as a gram of gallic acid equivalent per liter of kombucha solution, g GAE/L);

Values with the different capital letters within a column are significantly different at *p* < 0.05, while the values with the different lowercase letters within a row are significantly different at *p* < 0.05 based on LSD analysis.

Flavonoids are ubiquitous in polyphenol compounds and are among the most abundant and widespread plant secondary metabolites. Many edible plants, including tea and pineapple, contain large amounts of flavonoids^61,82,87^. The flavonoid contents of the kombuchas produced from black tea and from fusions of black tea and PPC were determined in this study, and the results are summarized in Table 5. The black tea kombucha (K1) exhibited higher flavonoid content than those produced from a fusion of black tea and the PPC. Increasing the PPC supplementation ratios led to a reduction in the initial flavonoid content of the kombucha, particularly for those supplemented with 40–60 g/L PPC; the initial flavonoid contents ranged from 21.68 to 24.05 mg QE/L. A marked decline in flavonoid content was observed during fermentation of the kombucha products supplemented with 10 g/L (K2), 20 g/L (K3), and 30 g/L PPC (K4), while other kombucha products (K1, K6, and K7) showed slight differences between the initial and final flavonoid contents. The changes seen in the flavonoid contents during fermentation may be attributable to oxidative reactions of the polyphenols, which was also reported by Gaggìa et al.^88^ and Jakubczyk et al.^7^ Fermentative biodegradation of the flavonoids into smaller molecules by the hydrolytic enzymes released from the microbial community in the kombucha SCOBY is also a possibility^89^. One example is that of LAB, especially *Lactiplantibacillus plantarum*, which releases β-glucosidase to degrade polyphenols and flavonoids^90,91^. In addition, other factors may also be involved, such as extractable reductions and esterification reactions occurring during fermentation^92^.

**Table 5:** Total flavonoid contents of kombucha products made from black tea and fusions of black tea and PPC.

Time (Day)	Kombucha products						
	K1	K2	K3	K4	K5	K6	K7
0	33.03 ± 0.46^Ed^	36.15 ± 0.35^Aa^	33.60 ± 0.22^Ac^	35.12 ± 0.15^Ab^	24.05 ± 0.30^Be^	23.87 ± 0.03^Be^	21.68 ± 0.10^Bf^
2	32.15 ± 0.02^Fa^	22.03 ± 0.12^Dd^	20.15 ± 0.12^Ff^	19.12 ± 0.05^Dg^	23.12 ± 0.23^CDb^	22.69 ± 0.25^Ec^	20.98 ± 0.05^Ce^
4	34.61 ± 0.28^ Da^	23.08 ± 0.09^Cd^	21.74 ± 0.13^De^	21.38 ± 0.16^Ce^	27.26 ± 0.29^Ab^	26.87 ± 0.26^Ac^	21.67 ± 0.14^Be^
6	35.53 ± 0.13^Ca^	23.11 ± 0.09^Cc^	21.90 ± 0.17^De^	21.40 ± 0.13^Cg^	23.40 ± 0.04^Cb^	22.52 ± 0.08^Ed^	21.67 ± 0.05^Bf^
8	35.92 ± 0.01^Ba^	23.36 ± 0.23^Cc^	22.05 ± 0.34^Dd^	21.35 ± 0.14^Ce^	24.52 ± 0.39^Bb^	23.62 ± 0.04^BCc^	21.87 ± 0.13^Bd^
10	35.54 ± 0.19^Ca^	22.31 ± 0.15^Dc^	21.27 ± 0.15^Ed^	21.24 ± 0.06^Cd^	23.45 ± 0.69^Cb^	23.21 ± 0.14^Db^	19.78 ± 0.29^De^
12	36.64 ± 0.10^Aa^	24.74 ± 0.21^Bb^	23.42 ± 0.12^Cc^	22.51 ± 0.62^Bd^	22.74 ± 0.04^Dd^	23.46 ± 0.24^CDc^	21.63 ± 0.02^Be^
14	33.24 ± 0.09^Ea^	24.99 ± 0.08^Bb^	23.91 ± 0.18^Bc^	22.81 ± 0.59^Be^	22.64 ± 0.05^De^	23.47 ± 0.16^CDd^	22.96 ± 0.07^Ae^

Data are mean ± SD (expressed as milligram of quercetin equivalent per liters of kombucha solution, mg QE/L);

Values with the different capital letters within a column are significantly different at *p* < 0.05, while the values with the different lowercase letters within a row are significantly different at *p* < 0.05 based on LSD analysis.

Most of the kombucha products studied here contained low levels of ethanol, ranging from 0.09 to 0.99% (v/v). The highest ethanol level was detected in the K3 kombucha product, while the lowest was found in the control kombucha (K1) (Table 6). It is noteworthy that supplementing the kombucha with PPC gave ethanol concentrations higher than that of the control kombucha made without PPC supplementation. The higher ethanol concentrations in the kombuchas supplemented with PPC may be correlated with the beneficial substances in the PPC since it contained not only sugar but also minerals and vitamins (Table 2), which could promote the growth and metabolic activity of the microbial community in the kombucha SCOBY. Similar results were also observed for kombucha fermented using fruit juices, such as apples, pomegranates, red grapes, and sour cherry juices, in which the final ethanol concentrations were 0.82%, 0.84%, 0.59%, and 0.67% (v/v) ethanol, respectively^93^. Compared with the current study, low values (0.30–0.60%)^94,95^ and high values for the ethanol content (1.14–5.83%)^7,88,96^ were also reported, which could be due to the differences in the raw materials used, the fermentation conditions, the sources and microbial compositions in the kombucha SCOBY, and the kombucha supplementing materials^97^. Regarding the health benefits of the kombucha, a low level of ethanol has a harmless influence on human health, similar to low-alcohol beverages in which the ethanol contents range from 0.05 to 1.20%^93^.

**Table 6:** Ethanol concentrations and total acidities of kombucha products after 14 days of fermentation.

Kombucha products	Ethanol concentration % (v/v)	Total acidity (g/L)
K1	0.09 ± 0.00^ g^	6.19 ± 0.08^f^
K2	0.38 ± 0.00^f^	19.88 ± 0.09^d^
K3	0.99 ± 0.01^a^	15.43 ± 0.26^e^
K4	0.65 ± 0.00^b^	23.72 ± 0.43^c^
K5	0.53 ± 0.01^d^	41.25 ± 0.09^a^
K6	0.55 ± 0.00^c^	32.79 ± 0.51^b^
K7	0.47 ± 0.00^e^	15.49 ± 0.17^e^

Results are presented as mean ± SD of three independent experiments.

The values with different letters within a column are significantly different at *p* < 0.05 based on LSD analysis.

During fermentation, yeast and bacteria in the kombucha SCOBY use different metabolic pathways to metabolize complex sugars into several beneficial substances, including organic acids. Many organic acids have been found in kombucha products, such as acetic acid, lactic acid, gluconic acid, glucuronic acid, citric acid, succinic acid, and butyric acid^5^, which influence the kombucha aroma and flavor. This accumulation of organic acids causes different changes in the total acidities and pHs of the kombucha products. The total acidities of kombucha products produced from the fusion of black tea and PPC were found to be in the range of 15.43‒41.25 g/L, which was higher than that of the control kombucha (6.19 g/L) (Table 6). As with the ethanol profiles, the increased accumulation of organic acids in the kombucha products supplemented with PPC might be correlated with the valuable substances in PPC that favor the growth and metabolic activity of the microbial community in the kombucha SCOBY. Previous studies also demonstrated that supplementing with beneficial substrates, such as lemon balm, mangosteen peel, spinach, apple, red grape, and pomegranate juice, enriched the kombucha SCOBY with much higher acid contents compared to traditional kombucha^93,98^. Notably, the organic acids accumulated in the kombucha products depended on several factors, such as the initial sugar content, the microbial composition of the kombucha SCOBY, and the fermentation conditions. Excess accumulation of organic acids with low pHs may adversely affect human health, such as acidosis; thus, the kombucha fermentation conditions should be optimized^14,15^.

### Volatile compounds in kombucha products

Based on GC–MS analyses, a total of 28 volatile compounds, including 12 esters, 6 carboxylic acids, 5 phenols, 3 alcohols, 1 aldehyde, and 1 ketone, were produced within 14 days of kombucha fermentation (Table 7), consistent with a report by de Melo et al.^99^ Other volatile compounds that were not detected in our study, such as amines, hydrocarbon lactones, and terpenes, were also reported by de Melo et al.^99^ The differences in the volatile compounds arising from different kombucha products may be associated with the raw materials used, the microbial composition of the kombucha SCOBY, and the fermentation conditions. Nineteen volatile compounds, mostly esters, carboxylic acids, and alcohols, were detected in the control kombucha (K1). In comparison, kombucha products made from fusions of black tea and the PPC (K2-K7) contained 19 to 23 compounds, of which the K7 kombucha product had the highest number of volatile compounds. Notably, 12 compounds, including ethyl acetate, ethyl decanoate, ethyl dodecanoate, acetic acid, isobutyric acid, 2-methylhexanoic acid, 4-ethyl-2-methoxyphenol, 4-ethylphenol, 4-(1,1-dimethylpropyl)phenol, ethanol, 2-methyl-1-butanol, and phenylethyl alcohol, were detected in all kombucha products. However, nine volatile compounds, including 2-methyl-2-methylbutyl butanoate, ethyl benzeneacetate, 2-phenethyl acetate, ethyl hexadecanoate, ethyl oleate, 2-methoxy-4-propylphenol, 4-(2-propenyl)phenol, benzaldehyde, and acetoin, were not detected in the control kombucha but were present in the kombucha products supplemented with the PPC, suggesting that these compounds may have been brought to the kombucha products during the fermentation process by raw material.

**Table 7:** Volatile compounds detected in different kombucha samples after 14 days of fermentation.

Volatile compounds	% area*							
	R_T_	K1	K2	K3	K4	K5	K6	K7
Esters								
Ethyl acetate	6.19	5.05	6.60	8.75	4.80	2.90	3.64	3.05
Butanoic acid, 2-methyl-, ethyl ester	10.34	1.50	n.d	n.d	n.d	n.d	n.d	0.91
Ethyl hexanoate	17.59	0.85	n.d	n.d	n.d	n.d	n.d	0.11
2-methyl-2-methylbutyl butanoate	19.37	n.d	n.d	n.d	n.d	n.d	n.d	0.21
Ethyl decanoate	34.83	2.46	1.88	3.44	3.34	2.94	4.89	5.07
Ethyl benzeneacetate	39.81	n.d	n.d	n.d	0.41	0.44	0.62	0.98
2-phenethyl acetate	40.90	n.d	n.d	2.92	2.37	1.78	1.86	3.85
Phenethyl acetate	40.99	2.32	4.10	n.d	n.d	n.d	n.d	n.d
Ethyl dodecanoate	42.43	2.75	1.51	3.02	2.92	2.78	3.68	4.09
Ethyl hexadecanoate	54.97	n.d	n.d	n.d	0.58	0.48	0.68	n.d
2-ethylhexyl salicylate	55.67	2.12	n.d	n.d	n.d	n.d	n.d	n.d
Ethyl oleate	59.63	n.d	n.d	n.d	0.43	0.20	0.50	0.79
Carboxylic acid								
Acetic acid	26.43	42.75	29.14	20.90	33.60	46.06	40.25	31.52
Isobutyric acid	31.49	0.65	0.52	0.41	0.41	0.47	0.60	0.78
2-methylhexanoic acid	35.47	5.31	2.79	2.08	1.82	1.89	2.63	3.61
Octanoic acid	49.30	3.89	1.12	1.21	0.62	n.d	0.73	0.55
n-Decanoic acid	54.92	6.05	1.80	1.41	0.59	n.d	1.02	1.37
Dodecanoic acid	59.46	3.58	1.08	0.74	n.d	n.d	n.d	n.d
Phenols								
4-ethyl-2-methoxy-phenol	48.12	3.32	17.75	24.16	23.39	20.23	19.45	22.12
2-methoxy-4-propyl-phenol	50.56	n.d	0.38	0.41	0.47	0.26	0.26	0.48
4-ethylphenol	52.26	7.04	13.61	12.13	9.08	7.16	6.22	7.10
4-(2-propenyl)-phenol	56.10	n.d	0.33	0.34	0.34	0.23	0.26	0.46
4-(1,1-dimethylpropyl)phenol	57.65	2.69	1.33	1.00	0.68	0.44	0.26	0.30
Alcohols								
Ethanol	7.25	4.74	5.79	7.35	7.56	7.64	8.24	7.06
2-methyl-1-butanol	16.50	0.95	0.99	0.98	0.80	0.56	0.72	0.88
Phenylethyl alcohol	44.26	1.99	7.95	8.07	5.44	3.18	3.00	3.38
Aldehydes								
Benzaldehyde	29.56	n.d	1.42	0.67	0.35	0.24	0.34	0.74
Ketone								
Acetoin	20.02	n.d	n.d	n.d	n.d	0.13	0.16	n.d

*Number expresses as % formulated from area detected of all compounds in kombuchas by GC–MS. *n.d.*, not detected.

A higher ester content indicates the superior quality of the beverage. Among esters, ethyl acetate, ethyl decanoate, and ethyl dodecanoate were the most abundant compounds detected in all kombucha products. These volatile compounds are commonly detected not only in kombucha products but also in many other alcoholic beverages, such as wines, spirits, and fruit distillates^100–104^. Ethyl acetate, a short-chain ester, is a flavor compound with a fruity and solvent-like aroma. Previous studies demonstrated that lipase-catalyzed esterification of acetic acid with ethanol yielded ethyl acetate as the main product^105^. In addition, various yeasts can also esterify acyl-CoA with ethanol to form ethyl acetate through alcohol acetyltransferases^106^. Ethyl acetate is found widely in various beverages, such as distilled beverages produced from coffee pulp^103^, peach spirits^104^, and vinegar made from a pineapple byproduct^102^. Ethyl decanoate, also known as ethyl caprate, is a fatty acid ester formed from decanoic acid or capric acid and ethanol. It contributes fruity and floral aromas and is frequently produced during winemaking, specifically at temperatures above 15 °C^107^. Other products, such as distilled beverages from coffee pulp and peach spirits, also contain high contents of ethyl decanoate^103,104^. Ethyl dodecanoate, a medium-chain ethyl ester, is one of the fermentation-derived esters commonly found in wine. It imparts floral and fruity hints to wine aromas^108,109^. The formation of ethyl dodecanoate was possibly correlated with the transformations of acyl-CoA caused by responding enzymes^109^. Ethyl dodecanoate is present not only in wine and kombucha but also in other beverages, such as distilled beverages made from coffee pulp and peach spirits^103,104^.

In addition to ethyl esters, three volatile acids, acetic acid, isobutyric acid, and 2-methylhexanoic acid, which mainly contributed to the acidic aroma, were detected in all kombucha products. Acetic acid is a predominant volatile acid with various beneficial effects on digestion, antioxidant levels, and lower lipid contents, and it regulates blood pressure^110,111^. It confers a vinegary flavor to the kombucha products^112^, and its production is mainly associated with AAB during kombucha fermentation using ethanol as a precursor. Other bacteria, such as *Blautia* sp. and *Faecalibacterium* sp.^70,71^, and some yeast genera, such as *Dekkera,* are also responsible for acetic acid production^73–75^. The concentrations of acetic acid detected in kombucha beverages varied from 7.65 to 18.00 g/L, depending on the raw materials used and the fermentation conditions^10^. Isobutyric acid, a typical alkyl carboxylic acid, is a short-chain fatty acid exhibiting both preventive and therapeutic potential in counteracting inflammation-mediated ulcerative colitis and colorectal cancer^113,114^. It is not widely found in kombucha beverages except in pollen-fermented kombucha^115^. Several bacteria are responsible for producing isobutyric acid, such as *Clostridium* sp., *Faecalibacterium* sp., and *Lactobacillus* sp.^116^ Methylhexanoic acid, also known as 2-methylcaproic acid, is a medium-chain fatty acid with a unique profile and a great combination of cheesy, creamy, and fruity flavors. It is naturally found in apples and strawberries. To the best of our knowledge, 2-methylhexanoic acid has not yet been discovered in kombucha beverages. Previous studies have demonstrated that this volatile acid is present in the Brazilian spirit Cachaca and in peach spirits^104,117^. Notably, other volatile acids, such as octanoic acid, decanoic acid, and dodecanoic acid, which are frequently associated with negative impacts on the sensory qualities of alcoholic beverages, were also found. These acids are associated with rancid and fat aromas^103,112^.

The major volatile phenols found in all kombucha products were 4-ethyl-2-methoxy-phenol, 4-ethylphenol, and 4-(1,1-dimethylpropyl)phenol. These volatile compounds are widely present in other fermented beverages, such as beer, cider, and wine, and their concentrations vary depending on the microbial compositions and fermentation conditions^118^. It has been previously reported that volatile phenols are possibly responsible for the flavors of kombucha beverages, and they exhibit unique profiles of barnyard, leather, smoke, and spice flavors^11,119,120^. Yeast, specifically *Dekkera*, and some LAB species, such as *Lpb. plantarum*, are responsible for volatile phenol production. These microbes convert the nonvolatile hydroxycinnamic acid, which is commonly found in fruits, vegetables, and grains, into volatile phenols, specifically 4-ethylphenol and 4-ethylguiaiacol, which affect the organoleptic properties of kombucha beverages^64,112,120^.

Alcohols also have positive effects on the aromas and flavors of kombucha. Three prominent alcohols were detected in all kombucha products: ethanol, 2-methyl-1-butanol, and phenylethyl alcohol. These alcohols are considered secondary products from yeast metabolism, and they contribute significantly to the aromas of alcoholic beverages^121^. Yeasts can synthesize these compounds through glycolysis or a catabolic pathway from the corresponding amino acids, such as valine, leucine, isoleucine, and phenylalanine^103^. The presence of phenylethyl alcohol in kombucha beverages has been reported to be associated with yeast activity, specifically that of *Zygosaccharomyces parabailii*^122^. It is one of the common macro components in beer and wine, exhibiting floral, rose, and honey scents^123^. It has recently been detected in kombuchas supplemented with hibiscus^99^. The branched-chain alcohol 2-methyl-1-butanol is one of the metabolites produced through the Ehrlich pathway, the biosynthetic pathways of fusel alcohols in yeasts^124^. This malt-tasting compound has also been detected in several plants, such as red raspberries, nectarines, carobs, black-eyed peas, garden cress, and horseradish trees. To our knowledge, it has not yet been found in kombucha beverages; however, this could be possible since the kombucha SCOBY contains several yeast species that may have metabolic activity in converting 2-keto acids into 2-methyl-1-butanol^124^.

The volatile compounds benzaldehyde and acetoin were found only in kombucha products made from a fusion of black tea and PPC. These compounds are commonly used as food additives to improve product flavors. Benzaldehyde imparts cherry and almond hints to the kombucha flavor, and its formation is closely associated with yeasts that use glycosidase enzymes, specifically *Z. parabailii*^122^. On the other hand, acetoin, which imparts a butter, creamy and cheese-like odor, is generated from the oxidation of lactic acid by some AAB species, such as *Acetobacter pasteurianus*^125^. Some LAB species and *Bacillus subtilis* also produce acetoin from pyruvate via α-acetolactate, and the enzymes responsible for acetoin formation are α-acetolactate synthase and α-acetolactate decarboxylase^126^. This volatile ketone has been found in Japanese sake, rice vinegar, and pineapple byproduct vinegar^102,127^.

### Antioxidant activities

**Figure 6:**
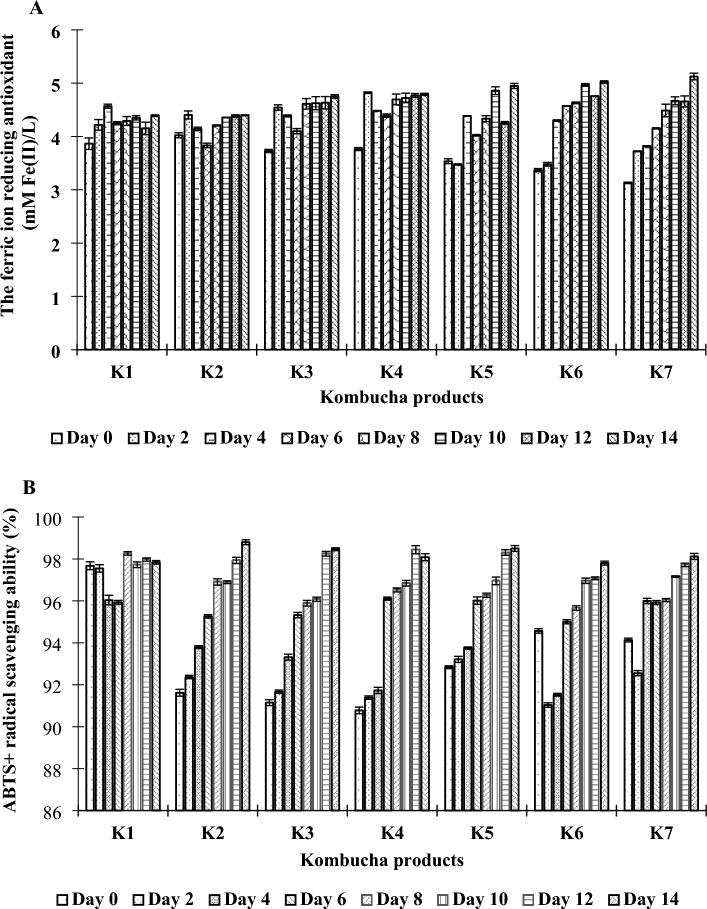
Antioxidant capacities of kombucha products made from black tea and fusions of black tea and PPC (as described in “Materials and Methods”) using FRAP (**A**) and ABTS (**B**) assays.

It has been reported that phenolics, flavonoids, and beneficial metabolites, such as organic acids, vitamins, minerals, and enzymes, generated during fermentation through the metabolic activities of the microbial community in the kombucha SCOBY play vital contributions to the antioxidant properties of the kombucha beverages^98,128^. Different antioxidants react with free radicals through different mechanisms, such as binding pro-oxidant metals, scavenging free radicals, and inhibiting pro-oxidant enzymes^129^. Therefore, various methods have been used to evaluate antioxidants, depending on their reaction mechanisms^130^. In this study, two different antioxidant activity platforms, the FRAP and ABTS + radical inhibition assays, were used to analyze the antioxidant activities of kombucha beverages. The results of the FRAP assay indicated that adding PPC into the fermentation medium, specifically at higher levels, decreased the reducing power of the kombucha. At day 0, the reducing power of the control kombucha was 3.87 mM Fe(II)/L, while values for the kombuchas supplemented with PPC were 3.13–3.73 mM Fe(II)/L, particularly for kombuchas K3 to K7. However, during fermentation, markedly increased antioxidant activities were detected in all of the kombucha products, and these were closely correlated with high TPCs, high total acidities, and low pHs. An increase in the antioxidant activity during fermentation was also reported by Chakravorty et al.^131^ Notably, at the end of fermentation (14 days), the kombucha beverages made from black tea and PPC exhibited the highest reducing powers, which were within the range of 4.40–5.12 mM Fe(II)/L; these were higher than that of the control kombucha (4.39 mM Fe(II)/L) (Fig. 6A).

Different kombucha products were tested as antioxidants scavenging the ABTS radical, and the results are summarized in Fig. 6B. The addition of PPC into the black teas (i.e., in K2, K3, K4, K5, K6, and K7) led to decreases in the antioxidant activities of the resulting kombuchas, as indicated by the FRAP assay. The proportion of the ABTS + radical scavenged by the control kombucha (day 0) was 97.68%, while the values for kombuchas made from fusions of black tea and PPC were 90.78–94.57%. Notably, the kombuchas made from the black tea and PPC exhibited considerable increases in their radical scavenging efficiencies during the fermentation process and reached maximum values within the range 97.80–98.80%, while the value for the control kombucha remained unchanged during fermentation. These results demonstrated that PPC enhanced the antioxidant activities of the kombucha beverages, consistent with previous studies on fruit juices, such as apple, pomegranate, and pineapple juice^33,132^, soursop^133^, olive leaf and honey^134^, and snake fruit^31,135^. Since the PPC contained several potential antioxidants, including polyphenols, minerals, and vitamins, as well as other beneficial substances that promoted the growth and metabolic activity of the microbial community in the kombucha SCOBY, those compounds, as well as the microbial metabolites generated during fermentation, i.e., organic acids and volatile compounds, may have contributed to the total antioxidant activities of the kombucha products.

### Antimicrobial activity

Kombucha has antimicrobial effects on various pathogenic microorganisms, including bacteria, yeasts, and fungi^10,20,136^. Several beneficial substances released from raw materials or generated through the metabolic activity of the microbial community during fermentation, such as polyphenols, organic acids (specifically acetic acid), alcohols, and other volatile compounds, have been attributed to the antimicrobial properties of kombuchas^5,6,8^. Different components have exhibited different mechanisms for preventing or inhibiting microbial growth. For instance, organic acids affect pathogenic and spore-forming bacteria by acidification of the cytoplasm and decreasing the intracellular pH^79,98^. Polyphenols can act in two ways: they can be modified into active forms to enhance beneficial bacteria or change their composition to inhibit pathogenic bacteria^137^. In this study, kombucha from fermented black tea and PPC exhibited vigorous antimicrobial activity against all tested pathogens, including gram-negative bacteria (*A. hydrophila, E. coli* ATCC 25922, *P. aeruginosa* ATCC 27853, and *S. typhi*) and gram-positive bacteria (*B. cereus*), similar to the control treatment using acetic acid (Table 8). These results of the current study are consistent with those reported by Cetajevic-Simin et al.^138^, who demonstrated that kombucha tea supplemented with lemon balm exhibited great antimicrobial activity against gram-negative bacteria, such as *P. aeruginosa* ATCC 27853, *Proteus mirabilis* ATCC 35659, *E. coli* ATCC 25922, *S. enteritidis* ATCC 13076, and *Erwinia carotovora* NCPPB 595, and gram-positive bacteria, such as *Staphylococcus aureus* ATCC 25923, *B. cereus* ATCC 10876, and *Sarcina lutea* ATCC 9341. Notably, no antimicrobial activity against any tested pathogen was detected in the control kombucha, possibly due to a low level of acetic acid in the control product. Another possibility is that the kombucha products made from black tea and PPC contained several volatile organic compounds that were not present in the control. These volatile organic compounds may have interacted with the bacterial cells and caused membrane damage, defective metabolism, and cellular disruptions that inhibited the growth of various microorganisms^139,140^. Moreover, many previous reports have demonstrated that the phenolic and flavonoid compounds released from the raw material limited bacterial growth^141–143^. Thus, the antimicrobial activities of kombucha beverages are due to the presence of organic acids and other bioactive components, such as polyphenols and volatile organic compounds. Therefore, black tea and PPC seem to be an effective combination for enhancing the antimicrobial activity of kombucha.

**Table 8:** Antimicrobial activities of kombucha products made from black tea and fusions of black tea and PPC.

Tested microorganisms	Acetic acid (pH 3.0)*	Kombucha products*						
		K1	K2	K3	K4	K5	K6	K7
*A. hydrophila*	+	−	+	+ +	+ +	+ +	+ + +	+ + +
*E. coli* (ATCC 25922)	+ +	−	+ +	+ +	+ + +	+ + +	+ + +	+ + +
*P. aeruginosa* (ATCC 27853)	+	−	+ + +	+ +	+ + +	+ + +	+ + +	+ + +
*S. typhi* DMST 22842	+	−	+ +	+ +	+ +	+ +	+ + +	+ + +
*B. cereus*	+	−	−	−	−	+	+	+

*Diameter of halo zone: − , no inhibition; + , 8–10 mm; +  + , 11–15 mm; +  +  + , 15–20 mm.

## Conclusion

Black tea kombucha made by using PPC as an alternative raw material to replace some or all of the sugar used in fermentation was produced in this study for the first time. Based on chemical composition analyses, the PPC contains several beneficial substances, such as sugars, organic acids, vitamins, minerals, and polyphenols, which could promote microbial growth, metabolic activity, and the biological properties of kombucha products. Supplementation with PPC enhanced the TPC, antioxidant, and antimicrobial properties of kombucha beverages against gram-negative and gram-positive bacteria. Several volatile compounds, including esters, carboxylic acids, phenols, alcohols, aldehydes, and ketones, which contributed to the flavors, aromas, and beneficial health effects, were detected in kombucha products made from black tea and PPC. These findings clearly demonstrated that PPC has ample potential for use as a supplement in producing functional kombucha beverages. Since numerous health benefits of kombucha are primarily determined using in vitro and in vivo assays using several human cell lines, there is no report on empirical evidence of the kombucha health benefits in human subjects. Therefore, further studies, specifically clinical trials, should be addressed.

### Submission declaration and verification

Submission of an article implies that the work described has not been published previously in any form.

## Data Availability

The DNA sequence datasets generated during the current study are available in the NCBI repository (https://www.ncbi.nlm.nih.gov/) with the accessions SRR23314099, SRR23314100, SRR23314101, and SRR23314102.
